# Insulin degrading enzyme contributes to the pathology in a mixed model of Type 2 diabetes and Alzheimer’s disease: possible mechanisms of IDE in T2D and AD

**DOI:** 10.1042/BSR20170862

**Published:** 2018-01-10

**Authors:** Huajie Li, Jian Wu, Linfeng Zhu, Luolin Sha, Song Yang, Jiang Wei, Lei Ji, Xiaochun Tang, Keshi Mao, Liping Cao, Ning Wei, Wei Xie, Zhilong Yang

**Affiliations:** 1 Department of Neurology, the First People’s Hospital of Changzhou, Jiangsu, China; 2 Department of General Laboratory, the First People’s Hospital of Changzhou, Jiangsu, China

**Keywords:** Alzheimer's disease, Insulin degrading enzyme, type 2 diabetes

## Abstract

Insulin degrading enzyme (IDE) is believed to act as a junction point of Type 2 diabetes (T2D) and Alzheimer's disease (AD); however, the underlying mechanism was not completely clear yet. Transgenic APPSwe/PS1 mice were used as the AD model and were treated with streptozocin/streptozotocin (STZ) to develop a mixed mice model presenting both AD and T2D. Morris Water Maze (MWM) and recognition task were performed to trace the cognitive function. The detection of fasting plasma glucose (FPG) and plasma insulin concentration, and oral glucose tolerance test (OGTT) were used to trace the metabolism evolution. Aβ40 and Aβ42 were quantified by colorimetric ELISA kits. The mRNA or protein expression levels were determined by quantitative real-time RT-PCR and Western blotting analysis respectively. T2D contributes to the AD progress by accelerating and worsening spatial learning and recognition impairments. Metabolic parameters and glucose tolerance were significantly changed in the presence of the AD and T2D. The expression levels of IDE, PPARγ, and AMPK were down-regulated in mice with AD and T2D. PPARγ activator rosiglitazone (RSZ) or AMPK activator AICAR increased the expression level of IDE and decreased Aβ levels in mice with AD and T2D. RSZ or AICAR treatment also alleviated the spatial learning and recognition impairments in AD and T2D mice. Our results found that, in the mice with T2D and AD, the activators of PPARγ/AMPK signaling pathway significantly increased the expression level of IDE, and decreased the accumulation of Aβ40 and Aβ42, as well as alleviated the spatial learning and recognition impairments.

## Introduction

Alzheimer’s disease (AD) is the most common cause of dementia in elderly people, accounting for approximately 70–90% of all cases [1]. No effective treatments are available for delaying the actual AD process since current treatment options only address symptoms [2]. Currently, one well-accepted hypothesis is that the main culprit of the pathogenesis of AD is the accumulation of Aβ in the brain, leading to neuronal destruction and synapse disruption [2,3]. Thus, exploring proteins associated with the degrading or accumulation of Aβ is of great importance.

Increasing evidence has demonstrated that Type 2 diabetes (T2D) exerts a contributive role in the development of AD. For example, T2D was proved to be related to a higher risk of AD in a Japanese study of subjects aged 65 years and older [4]. A longitudinal study based on Netherlandish subjects (over 5000) aged 55 years and older without dementia at baseline was performed, the results found a higher risk of AD in people with T2D, importantly, this association was stronger in subjects with T2D who reported insulin treatment [5]. A recent study indicated that improving T2D metabolic control could delay or prevent AD pathology [6]. Impaired insulin secretion and resistance was the relevant link between T2D and AD, and the glucose intolerance also seems to be associated with increased risk of AD [6]. It seems that proteins associated with the regulation of insulin levels can strengthen the contributive role of T2D to the development of AD.

Insulin-degrading enzyme (IDE) is a protein that plays a key role in degrading Aβ monomer *in vitro* and *in vivo* [7]. IDE is also a metalloprotease enzyme responsible for insulin degradation [8]. Previous studies indicated that IDE is genetically associated with AD in the Finnish, Chinese, and Europen populations [9,10]. In addition, some linkage studies have identified chromosome 10q as a site of a number of markers for increased plasma levels of Aβ that are close to the IDE site [11–14]. These results demonstrated the important role of IDE in AD progress [15]. Particularly, IDE is also believed to act as a junction point of T2D and AD [16]. However, the underlying mechanism was not completely clear yet. A study has reported PPARγ can induce the expression of IDE [17]. Some studies have suggested that PPARγ interacts directly with AMPK γ subunits in yeast two-hybrid assays, indicating that PPARγ may be a target for regulation by AMPK [18,19]. Thus, it raises the possibility that PPARγ and AMPK may regulate IDE expression in mice with AD and T2D.

In our study, we developed a mixed mice model presenting both AD and T2D. The Morris Water Maze (MWM), recognition task, oral glucose tolerance test (OGTT) and the detection of fasting plasma glucose (FPG), and plasma insulin concentration were used to validate the successful construction of AD and T2D mice model. Aβ40 and Aβ42 were quantified and the mRNA or protein expression levels were determined. Our study found that, the expression level of IDE was decreased in mice with T2D and AD. However, after treatment of the activators of PPARγ/AMPK signaling pathway, the expression level of IDE was increased significantly, the accumulation of Aβ40 and Aβ42 was decreased, and the activators also alleviated the spatial learning and recognition impairments in mice with T2D and AD.

## Materials and methods

### Animals

AβPPswe/PSEN1dE9 (Aβ PP/PS1) transgenic mice were used. The double-transgenic (Tg) mice were obtained from Jackson Laboratory (strain name B6C3-Tg (A βPPswe, PSEN1dE9) 85Dbo/J; stock number 004462). These B6C3-based Tg mice harbor two AD-related genes, one encoding a chimeric mouse/human amyloid-β protein precursor containing the K595N/M596L Swedish mutation (Aβ PPswe) and the other a mutant human presenilin 1 carrying a deletion of exon 9 (PSEN1dE9). APPSwe is the Swedish mutation of the amyloid precursor protein, and PS1 is the mutant form of human presenilin 1 [20]. The mice were genotyped from tail tissue as previously described [21]. Animals were maintained at room temperature (25 ± 2 °C) under a controlled environment (12h-light/12h-dark cycle), with free access to water and food. Procedures were approved by the First People’s Hospital of Chang Zhou.

### Induction of T2D mice

To create the T2D–AD model mice, after fasting overnight for 12–14 h, AβPP/PS1 Tg mice (age of 4.0 months) were received a single intraperitoneal injection of streptozocin/streptozotocin (STZ) as previously described (50 mg/kg; Sigma-Aldrich, St Louis, MO, U.S.A.) for 5 days [22], which is widely used in the induction of T2D mice model [23]. STZ solution was prepared by dissolving it in 0.1 M citrate buffer (pH 5.5) and was terminally sterile-filtered. Mice with fasting blood glucose levels above 12 mmol/l and higher insulin levels were considered T2D mice. At age of 6 months, the mice were enrolled for MWM and recognition task. Twenty-four hours later, the blood samples were collected via the tail vein method and metabolic measurements were performed. Finally, mice were anesthetized and killed and brain samples were collected for analysis. Procedures were approved by the Ethical Committee of the First People’s Hospital of Chang Zhou.

### Treatments

Mice were divided into six groups: Control, STZ, APP/PS1, APP/PS1 + STZ, APP/PS1 + STZ + RSZ, and APP/PS1 + STZ+ AICAR group (*n* = 10 in each group). Mice with wide-type littermates were used as a normal aging control (Control group). Wide-type littermates that received a single intraperitoneal injection of 50 mg/kg STZ were named as STZ group. AβPP/PS1 Tg mice (APP/PS1 group) that received a single intraperitoneal injection of 50 mg/kg STZ were named as APP/PS1 + STZ group. To determine the effects of PPARγ and AMPK on the expression levels of IDE, the induced T2D and AβPP/PS1 Tg mice were received a subcutaneous injection of PPARγ activator RSZ (5 mg/kg; Sta. Cruz Biotechnology, Santa Cruz, CA) (APP/PS1 + STZ + RSZ group), or received an intraperitoneal injection of AMPK activator AICAR (500 mg/kg; Wako PureChemicals Industries Ltd, Osaka, Japan) (APP/PS1 + STZ + AICAR group). Procedures were approved by the First People’s Hospital of Chang Zhou.

### MWM and recognition task

MWM task was to used to evaluate the escape latency (the time to reach the hidden platform), traveled distance (the length of swim path), and times across platform, as previously described [24]. The experimental apparatus consists of a circular water tank (100 cm in diameter, 40 cm in height), containing water (23 ± 1°C) to a depth of 15.5 cm, which was opaque by adding white ink. A transparent escape platform (10 cm in diameter, 20 cm in height) was hidden 1 cm below the water surface and position at the midpoint of one quadrant. Each mice received training per day for 6 days using a single hidden platform in one quadrant with three quadrants of rotational starting. Latency to escape from the water maze (finding the submerged platform) was recorded for each trial. On day 7, mice were subjected to a probe test in which platform was removed and each mouse was permitted to swim freely for 60 s. All data were recorded using video tracking software (SMART, Panlab Harward Apparatus; Bioscience Company, Holliston, MA, U.S.A.).

Recognition task was preformed as described previously [25]. On experimental day, each group of mice was subjected to two sessions and each session was composed of one trial. During first session (acquisition trial, trial 1), mice were placed in the arena containing two identical objects for 5 min. Exploration is defined as the mice directing its nose within 1 cm of the object while sniffing, looking at, or touching it. Any mice not exploring the objects for 20 s within the 5-min period were excluded from experiments. The second session (retrieval session, trial 2), 2 h after trial 1, one of the objects presented in the first trial was replaced by an unknown object, mice were placed back in the arena for 5 min and total time spent in exploration of each object and locomotor activities were determined. Locomotor activity and time spent in active exploration of the familiar (F) or novel (N) object on trial 2 were measured using the video-based Ethovision System. Recognition memory was evaluated using a recognition index (RI) calculated for each mice using the formula: [(N − F)/(N + F)] × 100 corresponding to the difference between the time exploring the novel and the familiar object, corrected for total time exploring both objects.

### Metabolic measurements

To determine FPG, the blood samples were collected sequentially from the tail vein at different time points as indicated in figures and the glucose oxidase method was used. For OGTT, glucose (3 g/kg) was introduced orally, then the glucose concentration in blood was measured at intervals of 0, 10, 20, 30, 40, 50, 60, 70, 80, 90, 100, 110, and 120 min. Plasma insulin concentration was measured by ELISA.

### IDE activity assay

Brain tissue extracts were prepared as previous described [26], the right hemisphere cortex was isolated for IDE activity detection. IDE activity was assessed with the InnoZyme Insulysin/IDE Immunocapture Activity Assay Kit (Calbiochem, Merck Millipore, MA, U.S.A.) which was normalized to the value of control group and the relative IDE activity was shown in the present study.

### Aβ ELISA

The frozen right hippocampus was used for detection of soluble Aβ40 and Aβ42 with the colorimetric ELISA kits (Wako, Japan) as previously described [6]. Briefly, tissue was homogenized in RIPA buffer (Beyotime P0013C, Haimen, Jiangsu, China) and centrifuged. Supernatants were diluted and the resultant pellet was extracted with formic acid and then centrifuged to obtain the supernatants. Absorbance was measured spectrophotometrically at 450 nm (MQX200R2, Biotek instruments, Burlington VT, U.S.A.).

### Western blotting

Western blotting was performed as previously described [27]. In brief, the frozen right hippocampus was lysed in RIPA buffer containing 150 mM NaF, 2 mM sodium orthovanadate, and protease inhibitors (protease inhibitor mixture; Roche). Protein of total lysate (20 μg) was loaded and blotted. The membranes were incubated with primary antibodies anti-IDE (MMS-282R; 1:1000; Covance, U.K.), anti-PPARγ (81B8, Cell Signaling Technology, Beverly, MA, U.S.A.), and anti-AMPK (1:1000; Santa Cruz Biotechnology, CA, U.S.A.) overnight at 4˚C, and then reacted with HRP-conjugated secondary antibodies (1:1000; Santa Cruz Company) at room temperature for 1.5 h. The protein bands were detected by ECL and visualized by UVP Gel imaging system (Upland, CA). The band intensity was analyzed by AlphaEaseFC (version 4.0).

### Quantitative real-time RT-PCR

RNA was extracted from the frozen right hippocampus using Trizol reagent (Invitrogen). RNA was quantified using a NanoDrop spectrophotometer (Thermo Scientific). The cDNA templates were synthesized with the SuperScript III First-Strand Synthesis SuperMix. The following oligonucleotide sequences were used as primers: 5′-CAATACATTCAGAAGCTACGTG-3′ (forward) and 5′-CAGGGTATGGTGTTGCATCTT-3′ (reverse). For β-actin, the primers were: 5′-GTTTGAGACCTTCAACACCCCA-3′ (forward) and 5′-CGAAGTCTAGGGCAACATAGC-3′ (reverse). Real-time RT-PCR was performed by using a Taq-Man gene expression assay kit (Life Technologies).

### Statistics

All experiments were performed in duplicate and repeated three times. Data were analyzed using the program Prism (GraphPad Software, Inc., La Jolla, CA, U.S.A.). Data were expressed as means ± SEM. Data were analyzed by one-way or two-way ANOVA. Statistical significance was set as *P*<0.05.

## Results

### The MWM results and metabolic parameters of mice with AD and T2D

**Figure 1:**
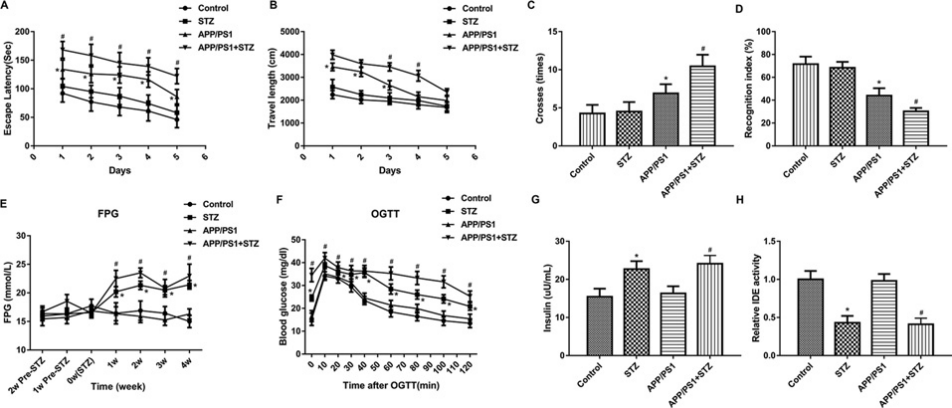
The MWM results and metabolic parameters of mice with AD and T2D The significant increases in escape latency (**A**), travel length (**B**), times cross the platform (**C**), and the significant decrease in recognition index (**D**) were found in mice with APP/PS1 transgenes and T2D as compared with mice that with APP/PS1 transgenes alone. (**E**) After STZ use, mice with T2D and AD showed markedly induction in FPG in comparison with AD mice. (**F**) The result of OGTT showed the significant induction of blood glucose level in T2D and AD mice as compared with AD mice. (**G**) The insulin level in T2D and AD mice was significant higher than that in mice with AD alone. (**H**) The activity of IDE in T2D and AD mice was significantly decreased as compared with that in the APP/PS1 group (*n* = 10 in each group, means ± SEM, ^*^P<0.05 compared with the control, and ^#^*P*<0.05 compared with the APP/PS1 group).

The presence of APP/PS1 transgenes caused significant increases in escape latency (Figure 1A), travel length (Figure 1B), times cross the platform (Figure 1C), and caused a significant decrease in recognition index (Figure 1D) in comparison with that in the control group. After STZ use, a significant increase in escape latency (Figure 1A), travel length (Figure 1B), times cross the platform (Figure 1C), and a significant decrease in recognition index (Figure 1D) were found in mice with APP/PS1 transgenes and T2D as compared with mice that with APP/PS1 transgenes alone. At the same time, the study found that STZ group had no effect on the cognitive function of mice. These results indicated the successful induction of AD, and suggested that T2D contributes to the AD progress by accelerating and worsening spatial learning and recognition functions.

Metabolic parameters and glucose tolerance were not altered in AD mice, but they were significantly changed in the presence of the AD and T2D. Figure 1E showed that the presence of APP/PS1 transgenes caused no alterations in FPG. But after STZ treatment, markedly induction in FPG in mice with T2D and AD was found in comparison with AD mice. The result of OGTT showed the significant induction of blood glucose level in T2D and AD mice as compared with AD mice, showing the impaired glucose tolerance (Figure 1F). Besides, the insulin level in T2D and AD mice was significant higher than that in mice with AD alone (Figure 1G). The results showed that FPG, OGTT, and insulin levels increased significantly when STZ was used alone compared with the control group. To some extent, the above results indicated the successful induction of T2D in AD mice. Besides, we found that the IDE activity reduced in STZ mice compared with the control group. Compared with the APP/PS1 group, the IDE activity of T2D and AD mice decreased significantly (Figure 1H).

### IDE was down-regulated in mice with AD and T2D

**Figure 2:**
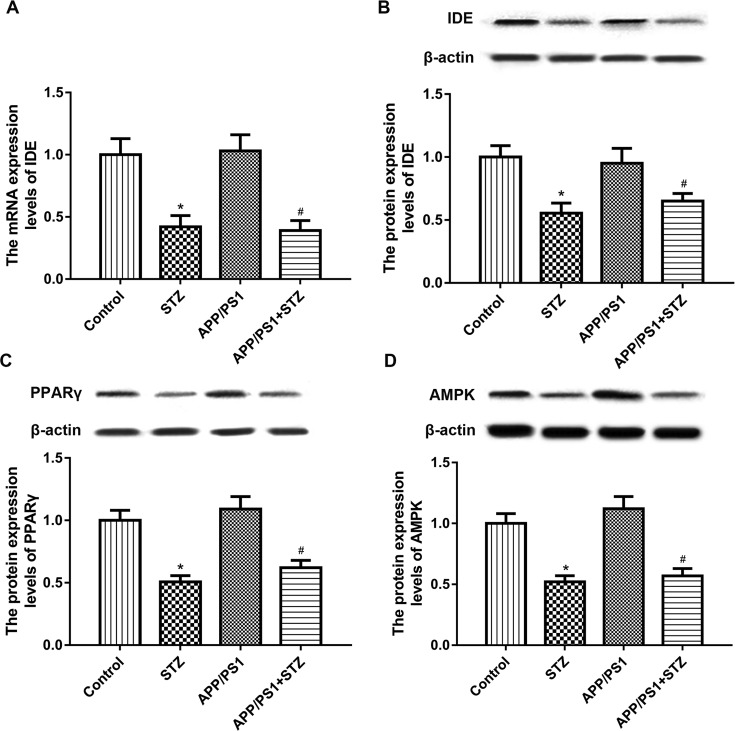
IDE, PPARγ, and AMPK were down-regulated in mice with AD and T2D (**A**) The mRNA expression level of IDE in AD and T2D mice was significant lower than that in AD mice. (**B**) The protein expression level of IDE in AD and T2D mice was significant lower than that in AD mice. (**C**) The protein expression level of PPARγ in AD and T2D mice was significant lower than that in AD mice. (**D**) The protein expression level of AMPK in AD and T2D mice was significant lower than that in AD mice (*n* = 10 in each group, means ± SEM, ^*^P<0.05 compared with the control, and ^#^*P*<0.05 compared with the APP/PS1 group).

We found that the mRNA expression level of IDE in AD mice showed no significant difference in comparison with that in control. However, the mRNA expression level of IDE in AD and T2D mice was significant lower than that in AD mice (Figure 2A). Consistently, the protein expression level of IDE in AD and T2D mice was significant lower than that in AD mice. Compared with the control group, the STZ group showed similar results (Figure 2B).

### PPARγ and AMPK were down-regulated in mice with AD and T2D

To further explore the underlying mechanism, we found that the protein expression level of PPARγ in AD mice showed no significant difference in comparison with that in control. However, the protein expression level of PPARγ in AD and T2D mice was significant lower than that in AD mice (Figure 2C). The protein expression level of AMPK in AD mice showed no significant difference in comparison with that in control. However, the protein expression level of AMPK in AD and T2D mice was significant lower than that in AD mice (Figure 2D). STZ alone also reduced the expression of PPARγ and AMPK compared with the control group.

### RSZ or AICAR increased the expression level of IDE and decreased Aβ levels in mice with AD and T2D

**Figure 3:**
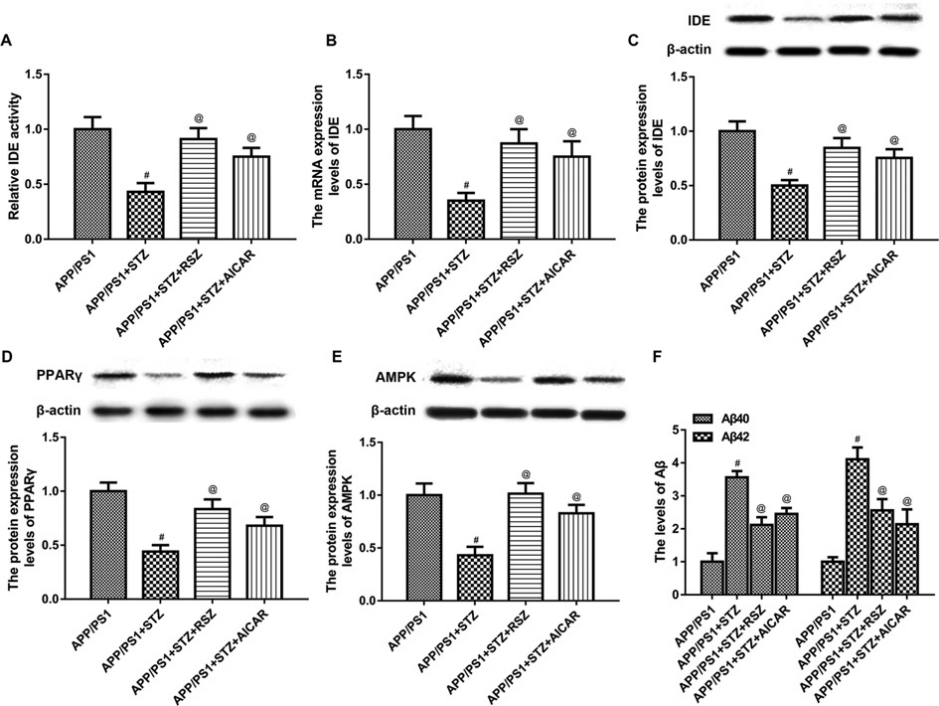
RSZ or AICAR increased the expression level of IDE and decreased Aβ levels in mice with AD and T2D (**A**) PPARγ activator RSZ or AMPK activator AICAR markedly increased the IDE activity. (**B**) PPARγ activator RSZ or AMPK activator AICAR markedly increased the mRNA expression level of IDE. (**C**) The protein expression level of IDE was also significantly increased after treatment of PPARγ activator RSZ or AMPK activator AICAR. (**D**) The protein expression level of PPARγ was also significantly increased after treatment of PPARγ activator RSZ or AMPK activator AICAR. PPARγ. (**E**) The protein expression level of AMPK was also significantly increased after treatment of PPARγ activator RSZ or AMPK activator AICAR. PPARγ. (**F**) After treatment with PPARγ activator RSZ or AMPK activator AICAR, the levels of soluble Aβ40 and Aβ42 were significantly decreased in mice with AD and T2D (*n* = 10 in each group, means ± SEM, ^#^*P*<0.05 compared with the APP/PS1 group, and ^@^*P*<0.05 compared with the APP/PS1 + STZ group).

PPARγ activator RSZ or AMPK activator AICAR was then use, the results revealed that the activity and mRNA expression level of IDE were markedly increased (Figure 3A and B). The protein expression level of IDE, PPARγ, and AMPK was also significantly increased after treatment of PPARγ activator RSZ or AMPK activator AICAR (Figure 3C–E). Besides, we found the levels of soluble Aβ40 and Aβ42 were significantly increased in mice with AD and T2D as compared with that in the control (Figure 3F). After treatment with PPARγ activator RSZ or AMPK activator AICAR, the levels of soluble Aβ40 and Aβ42 were significantly decreased.

### The MWM results of mice with AD and T2D after treatment with RSZ or AICAR

**Figure 4:**
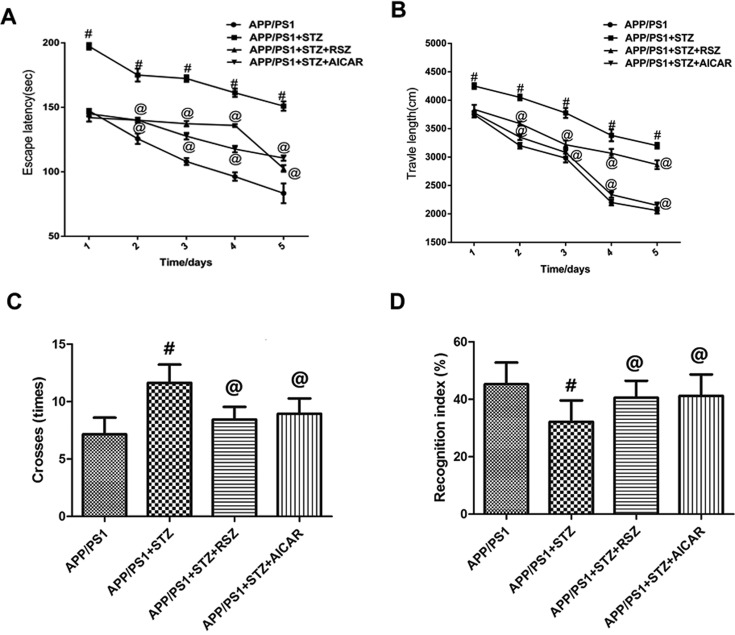
The MWM results of mice with AD and T2D after treatment with RSZ or AICAR Treatment with RSZ or AICAR in AD and T2D mice caused significant decreases in escape latency (**A**), travel length (**B**), times cross the platform (**C**), and caused a significant increase in recognition index (**D**) in AD and T2D mice (*n* = 10 in each group, means ± SEM, ^#^*P*<0.05 compared with the APP/PS1 group, and ^@^*P*<0.05 compared with the APP/PS1 + STZ group).

As shown in Figure 4, treatment with RSZ or AICAR in AD and T2D mice caused the significant decreases in escape latency (Figure 4A), travel length (Figure 4B), and times cross the platform (Figure 4C). However, after treatment with RSZ or AICAR, we also found the significant increase in recognition index in AD and T2D mice (Figure 4D). These results suggested that PPARγ activator RSZ or AMPK activator AICAR effectively alleviated the spatial learning and recognition impairments in T2D and AD mice.

## Discussion

In present study, we first developed an animal model presenting both AD and T2D. Metabolic parameters and glucose tolerance were not altered in AD mice, but they were significantly changed in the presence of the AD and T2D, indicating the successful induction of T2D in AD mice. The significant increase in escape latency, travel length, times cross the platform, and the significant decrease in recognition index were found in mice with AD and T2D as compared with mice overexpressing APP/PS1, indicating that T2D contributes to the AD progress by accelerating and worsening spatial learning and recognition impairments. Besides, we found the levels of soluble Aβ40 and Aβ42 were significantly increased in mice with AD and T2D as compared with that in the control. These results suggested that T2D contributes to the AD progress also by promoting the accumulation of Aβ. A previous study [28] summarized the results from eight population based cohort studies concluded that the associations between T2D and AD remain conflict. Of the studies meeting inclusion criteria, six showed a significantly increased relative risk of AD in patients with T2D. By contrast, two found no significant association between T2D and AD [27]. Our study may support the conclusion that T2D has a contribution role to the AD progress, the impaired spatial learning and recognition functions, as well as the accumulation of Aβ were consistent with a previous study [6].

The underlying mechanism was then explored. Most studies accepted that IDE is believed to act as a junction point of T2D and AD. However, a number of genetic studies concluded that IDE was not associated with late-onset AD [29,30]. We found that the expression level of IDE was significantly down-regulated in mice with AD and T2D as compared with that in AD mice. However, the expression level of IDE in AD mice showed no significant difference in comparison with that in control. These results may suggest the potential role of IDE in mice with T2D and AD. Chen et al. [31] indicated that the immunostaining of PPARγ was markedly down-regulated in STZ-induced diabetic mouse cartilages. Consistently, our results showed the down-regulated expression of PPARγ in T2D and AD mice. Studies have reported PPARγ can induce the expression of IDE, which may promote the removal of Aβ in the glial cells and neurons [17]. Further studies showed that PPARγ could regulate IDE expression in rat primary neurons through binding to a functional peroxisome proliferator-response element (PPRE) in IDE promoter and promoting IDE gene transcription [32]. AMPK is a regulator of energy homeostasis and is an evolutionarily conserved heterotrimer that consists of α-catalytic and β- and γ-regulatory subunits [33]. Some studies have suggested that PPARγ interacts directly with AMPKγ subunits in yeast two-hybrid assays, indicating that PPARγ may be a target for regulation by AMPK [18,19]. Here, we found the down-regulated expression of AMPK in T2D and AD mice. Importantly, AMPK is also considered to be a target for preventing T2D [33].

It was reported that PPARγ agonist RSZ can regulate brain IDE levels in an animal model of AD [15,17]. In our study, we found that RSZ or AICAR increased the expression level of IDE and decreased Aβ levels in mice with AD and T2D. Besides, treatment with RSZ or AICAR in AD and T2D mice caused the significant decreases in escape latency, travel length, times cross the platform, but resulted in the significant increase in recognition index. Consistently, Xiang et al. [34] indicated that PPARγ agonist, pioglitazone, exerts a significant neuroprotective effect against scopolamine-induced cholinergic system deficit and cognitive impairment by significantly decreasing the escape latency. These results suggested that PPARγ activator RSZ or AMPK activator AICAR effectively increased the expression level of IDE, decreased the accumulation of Aβ40 and Aβ42, and alleviated the spatial learning and recognition impairments in T2D and AD mice.

Combine previous reporting [35] and the present study, we found the potential role of IDE may as a target for AD with metabolic perturbations. Especially when AD patients present with peripheral and central metabolic impairments. Our research suggests that a new perspective, PPARγ agonist and AMPK activator may be a potential strategy for treatment of AD.

Totally, our results indicated that, in the mice with T2D and AD, the activators of PPARγ/AMPK signaling pathway significantly increased the expression level of IDE, and decreased the accumulation of Aβ40 and Aβ42, as well as alleviated the spatial learning and recognition impairments. To some extent, PPARγ and AMPK activators may be potential therapeutic approaches for patients with T2D and AD.

## Abbreviations

AD: Alzheimer’s disease
FPG: fasting plasma glucose
IDE: insulin degrading enzyme
MWM: Morris Water Maze
OGTT: oral glucose tolerance test
PPRE: peroxisome proliferator-response element
RSZ: rosiglitazone
STZ: streptozocin/streptozotocin
T2D: Type 2 diabetes
RT-PCR: reverse transcription-polymerase chain reaction
AMPK: AMP activated protein kinase
PPARγ: peroxisome proliferator-activated receptor γ
